# A Laser‐Induced Chrysiasis Treated With Pulsed Intense Light and Chemical Peels

**DOI:** 10.1111/jocd.71005

**Published:** 2026-07-14

**Authors:** Rosemarie Mazzuco, Juliano Adolfo Grock, Airá Novello Villar, Sarah Lyane Venzon, Analupe Webber

**Affiliations:** ^1^ Private Clinic at Éclat Dermatologie by Rosemarie Mazzuco Balneário Camboriú Brazil; ^2^ Private Practice Chapecó Brazil; ^3^ Institute of Dermatology Prof Rubem David Azulay (IDPRDA), Santa Casa de Misericórdia Rio de Janeiro Brazil; ^4^ Universidade da Fronteira Sul Passo Fundo Brazil; ^5^ Hospital São Lucas da Pontifícia Universidade Católica Do Rio Grande Do Sul Porto Alegre Brazil; ^6^ Derm4 Dermatological Clinic Porto Alegre Brazil; ^7^ Dermatology Department Pontifícia Universidade Católica Do Rio Grande Do Sul Porto Alegre Brazil

## Abstract

**Background:**

Gold salts (chrysotherapy) were widely used for the treatment of moderate to severe rheumatoid arthritis during the 1990s. Following administration, gold particles may deposit permanently in multiple tissues, including the skin. Exposure to ultraviolet radiation can induce a characteristic blue‐gray pigmentation known as chrysiasis. More recently, short‐ and ultra‐short‐pulse lasers have also been implicated in triggering chrysiasis, although the underlying mechanism remains poorly understood.

**Objective:**

To describe two cases of laser‐induced chrysiasis occurring decades after chrysotherapy and to propose a simple treatment approach using intense pulsed light (IPL) and chemical peels.

**Methods:**

Two patients with a remote history of gold salt therapy for rheumatoid arthritis, discontinued more than 30 years earlier, developed facial blue‐gray pigmentation following laser procedures. Clinical findings, treatment strategy, and outcomes were evaluated.

**Results:**

Both patients presented with laser‐induced chrysiasis affecting sun‐exposed facial areas. Treatment combining IPL sessions and chemical peels resulted in rapid and significant improvement in pigmentation, with good cosmetic outcomes and no major adverse effects.

**Conclusion:**

Laser‐induced chrysiasis may occur even decades after gold salt administration. Dermatologists should routinely investigate prior chrysotherapy exposure, particularly in patients with a history of rheumatoid arthritis, before performing laser treatments. IPL combined with chemical peels may represent a safe and effective therapeutic option for managing this challenging condition.

**Abbreviations**
IPL: intense pulsed light
TCA: trichloroacetic acid

## 1 Introduction

Gold salts (chrysotherapy) were the standard treatment for moderate to severe rheumatoid arthritis from the 1920s until the late 1980s, when methotrexate became the first‐line drug due to greater efficacy, faster onset, and a more favorable safety profile [1].

The mechanism of action of gold salts is not fully understood; however, once administered orally or parenterally, these salts deposit in organs and tissues such as the liver, kidneys, reticuloendothelial system, bone marrow, and skin, where they may remain indefinitely [2]. Chrysiasis is an example of persistent, sometimes permanent, gold deposition in tissues [3], manifesting as blue‐gray, photoinduced cutaneous pigmentation whose intensity correlates with cumulative gold dose. Laser‐induced chrysiasis was first described by Trotter et al. [4] as a rare complication occurring even decades after gold therapy cessation.

Since the late 1990s, nanosecond Q‐switched lasers (Ruby, Alexandrite, Nd:YAG) have been increasingly used in dermatology for pigmented lesions, tattoo removal, and photorejuvenation. Picosecond lasers have gained popularity since the 2010s for similar indications, with superior efficacy [5].

We present two cases of chrysiasis triggered by Q‐switched and picosecond lasers in Caucasian patients treated with gold salts more than 30 years earlier.

## 2 Case Reports

### 2.1 Patient 1

**FIGURE 1:**
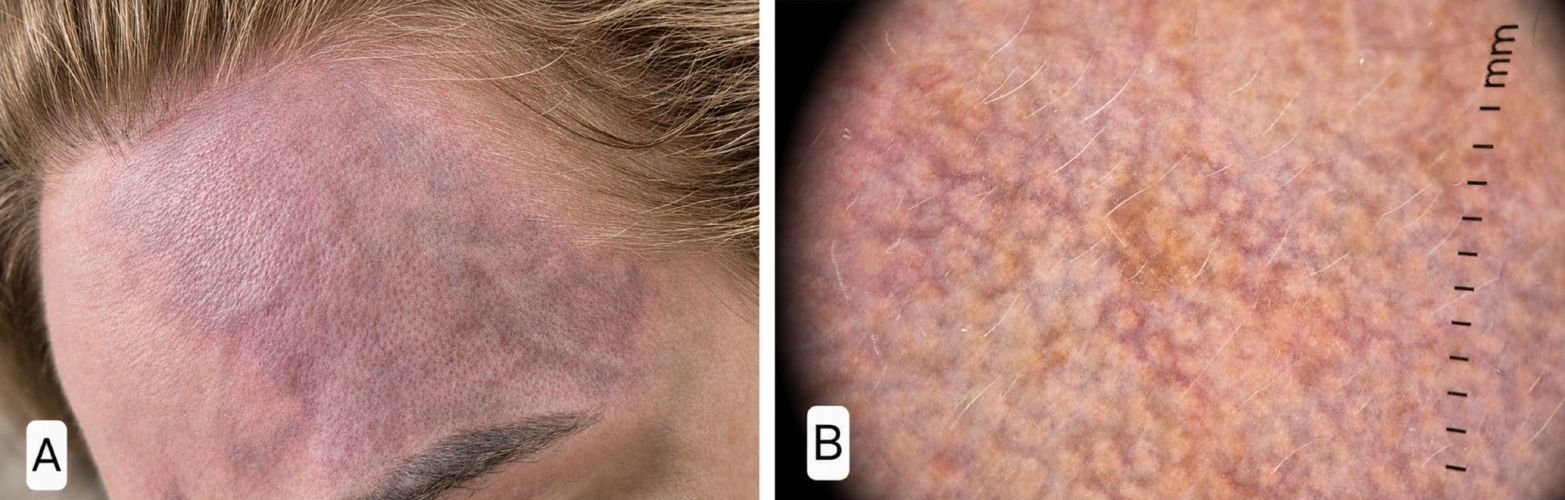
(A) Erythema, edema, and darkening of the area treated with Q‐switched Nd:YAG laser, immediately after the procedure. (B) Dermoscopy of the affected area showing blue‐violet pigmentation with a reticulated pattern surrounding the pilosebaceous units.

A 60‐year‐old woman with juvenile rheumatoid arthritis received intramuscular gold salts (aurothioglucose 5%; Solganal, Schering Plow, Kenilworth, NJ) from 1989 to 1994 (31 years prior). She currently uses rituximab with good disease control and no prior clinical evidence of chrysiasis. In April 2025, she underwent 1064 nm Q‐switched Nd:YAG laser photorejuvenation. During the laser application, she experienced intense local pain followed by erythema, edema, and violaceous pigmentation (Figure 1A). Dermatoscopy revealed blue‐black pigment distributed in a reticular pattern around pilosebaceous units (Figure 1B).

**FIGURE 2:**
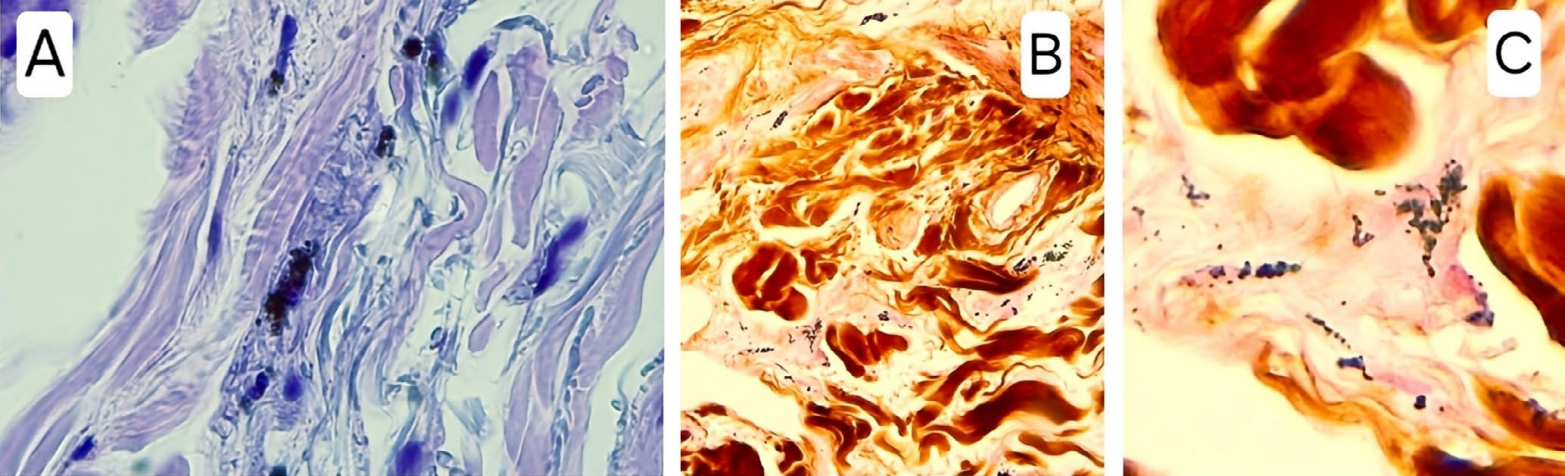
(A) Hematoxylin and eosin (H&E), 400×: Finely granular deposits of refractile brownish material are observed predominantly in the papillary and reticular dermis. These deposits are present both within the cytoplasm of dermal macrophages and freely in the extracellular matrix, located between collagen and elastic fibers. There is no involvement of the epidermis. A mild interstitial inflammatory infiltrate is present, composed of scattered lymphocytes and macrophages, without granuloma formation. (B, C) Grocott's methenamine silver (GMS) stain, 400×: The stain highlights metallic deposits as black‐stained, refractile granules distributed in the extracellular matrix and phagocytosed by dermal macrophages. This staining likely represents the adsorption of silver to the surface of the fragments following fragmentation, photothermolysis, and oxidation reactions.

Inflammatory signs resolved within hours; however, grayish‐blue discoloration persisted. Laser‐induced chrysiasis was suspected. Histopathology with hematoxylin and eosin (H&E) staining showed brownish‐black granules and rounded structures in the superficial dermis, distributed interstitially, perivascularly, and within macrophage cytoplasm (Figure 2A). These deposits stained positively with Grocott's methenamine silver stain (Figure 2B,C) but were negative for Perl's (hemosiderin) and Fontana‐Masson (melanin) stains.

**FIGURE 3:**
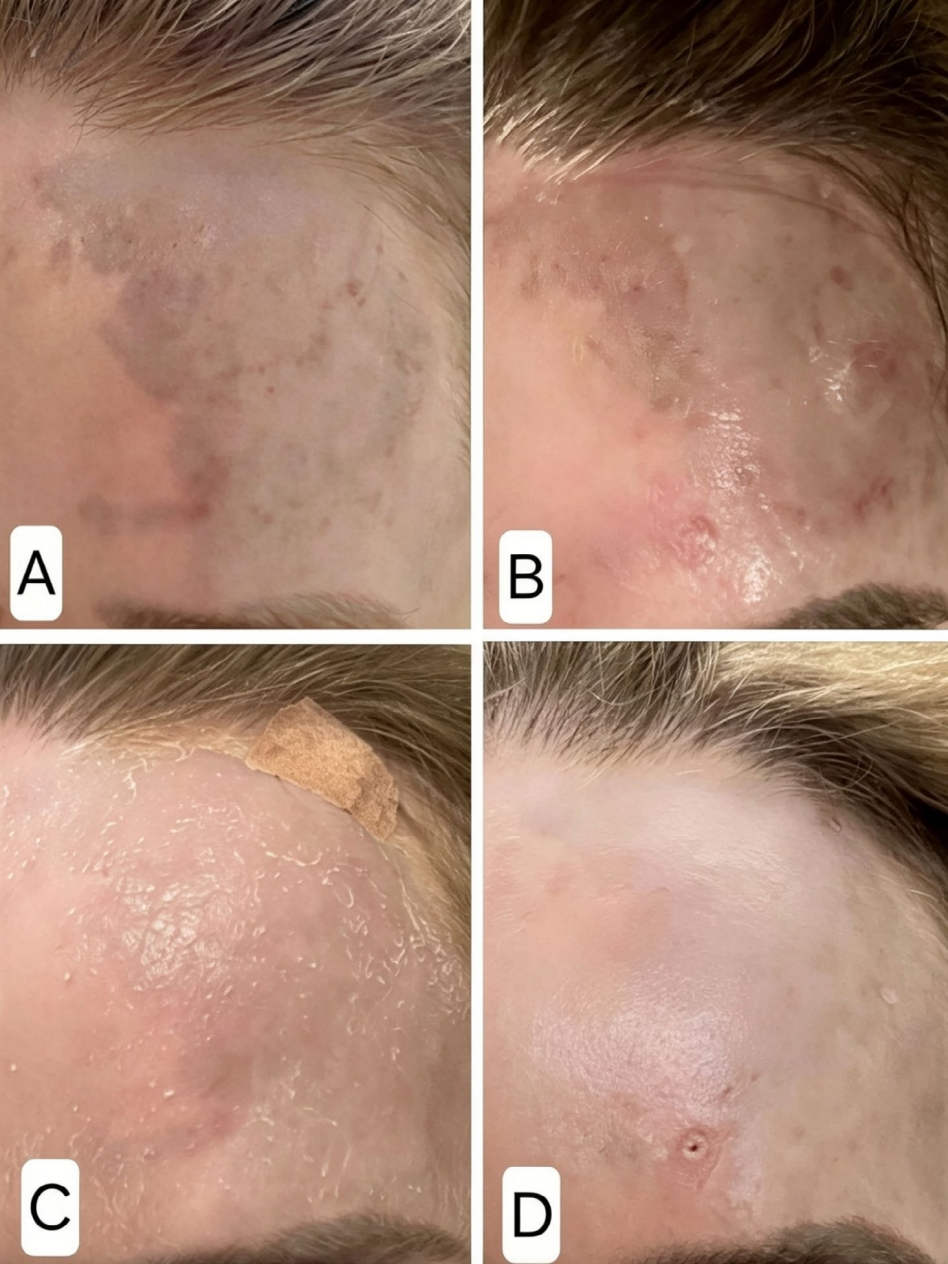
(A) Blue‐gray pigmentation, consistent with laser‐induced chrysiasis, in the area treated with 1064 nm Q‐switched Nd:YAG laser, 10 days after the procedure. (B) Same area, 5 days after the first session of IPL (695 nm) and 25% TCA peel, with residual crusts. (C) Same area, 8 days after the second session of IPL (695 nm) and 25% TCA peel. (D) Significant improvement in pigmentation after the fourth treatment session.

The patient underwent biweekly sessions of 695 nm Intense Pulsed Light (IPL; 15 J/cm^2^, 5 ms; Etherea MX, Vydence Medical, São Carlos, Brazil) immediately followed by 25% trichloroacetic acid (TCA) peel applied only to pigmented areas. After four sessions, approximately 90% pigmentation reduction was observed clinically (Figure 3A–D).

### 2.2 Patient 2

**FIGURE 4:**
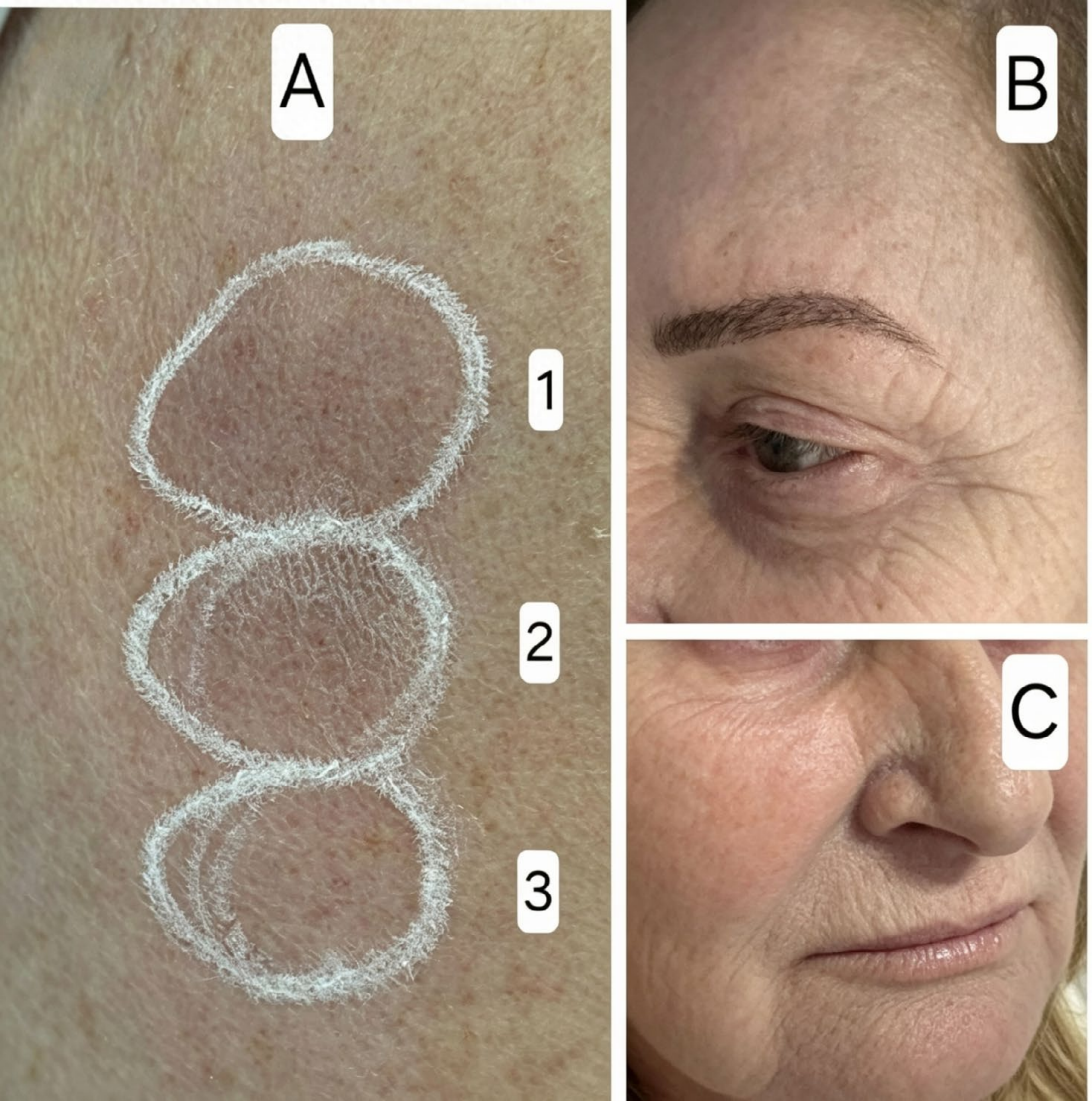
(A) Test sites on the arm of the 72‐year‐old patient: (1) 1064 nm collimated picosecond laser; (2) 532 nm collimated picosecond laser; (3) 1064 nm long‐pulse laser. Note that areas 1 and 2 developed similar dark pigmentation regardless of wavelength, while area 3 showed no pigmentation. (B, C) Diffuse grayish‐blue discoloration on the face, predominantly around the mouth, nose, and forehead, 24 h after full‐face picosecond laser treatment.

A 70‐year‐old woman with rheumatoid arthritis was treated with gold salts 36 years prior (regimen unknown), followed by corticosteroids, chloroquine, methotrexate, and currently naproxen, deflazacort, cannabidiol, and leflunomide for disease control. In March 2025, she received full‐face treatment with 1064 nm and 532 nm picosecond lasers. Within 24 h, grayish‐blue discoloration developed diffusely on the face, predominantly periorally, nasally, and frontally (Figure 4B,C).

Suspecting laser‐induced chrysiasis, three picosecond laser wavelengths were applied as test sites on the patient's arm (Figure 4A1–3). Sites treated with 1064 nm and 532 nm picosecond lasers developed similar dark pigmentation, while the 1064 nm long‐pulse laser site showed no pigmentation. Histopathology matched Patient 1's findings. Following diagnosis, she underwent four sessions of 695 nm IPL combined with 25% TCA peel. Moderate improvement was noted, but she wanted to stop the treatment.

## 3 Discussion

Chrysiasis is a rare dermatosis, typically appearing after a cumulative elemental gold dose of ≥ 20 mg/kg, predominantly in photoexposed areas [2]. Laser‐induced chrysiasis is extremely rare, first described by Trotter et al. in 1995 after Q‐switched ruby laser exposure [4]. Few subsequent cases triggered by Alexandrite and Nd:YAG Q‐switched lasers have been reported [6, 7, 8], with fewer than 10 cases in the literature to date.

The exact mechanism remains incompletely understood. It's hypothesized that laser energy absorbed by dermal gold deposits—despite gold not being a primary chromophore—induces photoacoustic or photomechanical effects fragmenting gold particles. This fragmentation alters light scattering, accentuating the Tyndall effect and producing the characteristic grayish‐blue pigmentation. Laser‐induced oxidation and redistribution of gold particles may also contribute [6].

Treatment has involved long‐pulsed lasers (ruby or dye), with or without fractional CO_2_ laser, but results are inconsistent [4, 9, 10]. In our cases, IPL with a 695 nm cutoff filter combined with 25% TCA peel—chosen for local availability and suitable wavelength—yielded superior clinical outcomes.

Two important points arise: (1) The latency between gold therapy and laser‐induced chrysiasis exceeded 30 years, indicating permanent dermal gold accumulation. Thus, a history of gold salt use must always be obtained before short‐ or ultra‐short‐pulsed laser treatments. (2) Despite rapid technological advances, simple procedures remain valuable for managing challenging dermatologic conditions.

## Author Contributions

Rosemarie Mazzuco – responsible for the design of the project, analysis of the clinical data, writing of the results and discussion sections, final review of the article. Juliano Adolfo Grock – responsible for providing clinical case data and reviewing the discussion. Airá Novello Villar – responsible for the examinations and anatomopathological data of clinical cases. Sarah Lyane Venzon – responsible for the literature review and preliminary writing of the article. Analupe Webber – responsible for the discussion of conducts, bibliographic review and follow‐up of clinical cases, assisting in the writing of results and conclusions.

## Funding

The authors have nothing to report.

## Ethics Statement

The authors have nothing to report.

## Consent

The authors obtained written consent from patients for their photographs and medical information to be published in print and online and with the understanding that this information may be publicly available. Patient consent forms were not provided to the journal but are retained by the authors.

## Conflicts of Interest

The authors declare no conflicts of interest.

## Data Availability

The data that support the findings of this study are available on request from the corresponding author. The data are not publicly available due to privacy or ethical restrictions.
